# Occult Renal Calcifications in Patients with Normocalcemic Primary Hyperparathyroidism and Their Association with the Parathyroid Hormone-Vitamin D Axis

**DOI:** 10.1155/2022/4558236

**Published:** 2022-04-08

**Authors:** Fernanda Victor, Alyne Layane Pereira Lemos, Anna Mirella de Holanda Ribas, Leonardo Bandeira, José Henrique Pimentel, Luiz Otávio de Andrade Damázio, Francisco Bandeira

**Affiliations:** ^1^Division of Endocrinology & Diabetes, and Division of Radiology, University of Pernambuco Medical School, Recife, Brazil; ^2^FBandeira Endocrine Institute, Recife, Brazil; ^3^Grupo Fleury, Recife, Brazil; ^4^Agamenon Magalhaes Hospital, University of Pernambuco Medical School, Recife, Brazil

## Abstract

Normocalcemic primary hyperparathyroidism (NPHPT) is characterized by elevated serum levels of parathyroid hormone (PTH) with persistently normal serum calcium concentrations after excluding secondary causes of hyperparathyroidism. Urolithiasis and/or nephrocalcinosis may occur in hypercalcemic PHPT, but little is known about these complications in NPHPT. *Objectives*. To identify occult urolithiasis and nephrocalcinosis in asymptomatic patients with NPHPT and evaluate biochemical markers as risk predictors for the development of renal calcification (RC). *Methods*. Cross-sectional analysis of 34 patients with no history of urolithiasis and/or nephrocalcinosis. The diagnosis of NPHPT was as follows: elevated serum PTH (reference range: 15–65 pg/mL), normal albumin-corrected serum calcium, normal urinary calcium excretion, serum 25(OH)D >30 ng/mL, eGFR (CKD-EPI) > 60 mL/min/1.73 m^2^, without intestinal disease, and not on medications such as thiazide diuretics, lithium, bisphosphonates, or denosumab. Patients were categorized according to the presence or absence of RC identified by renal imaging. Their clinical and biochemical characteristics were then compared. *Results*. The patients had a mean age of 67.97 ± 10.45 years, predominantly postmenopausal women (88.2%); serum PTH, 119.67 ± 64.44 pg/mL; 25(OH)D, 39.00 ± 8.88 ng/dL; 1.25(OH))_2_D, 74.53 ± 26.37 pg/mL; corrected serum calcium, 9.34 ± 0.62 mg/dL; and 24-hour urinary calcium, 134.87 ± 79.68 mg/day. RC was identified in 26.5% of the patients. There was no difference in anthropometric and clinical parameters, renal function, 25(OH)D, and urinary pH in patients with or without RC. Patients with RC had higher PTH values (176.22 vs. 99.32 pg/mL, *P* = 0.001), 1.25(OH) 2D (96.83 vs. 62.36 pg/mL, *P* = 0.005), and 24-hour urinary calcium (181.9 vs. 117.94 mg/day, *P* = 0.037). *Conclusion*. Occult renal calcifications are common in NPHPT and are associated with increased serum PTH, 1.25(OH))_2_D, and 24 h urinary calcium.

## 1. Introduction

Normocalcemic primary hyperparathyroidism (NPHPT) is a disorder of the calcium metabolism characterized by elevated serum parathyroid hormone (PTH) levels with persistently normal serum calcium concentrations [1, 2]. Secondary causes of hyperparathyroidism need to be excluded, such as medications known to affect PTH levels (diuretics, lithium, denosumab, bisphosphonates, anticonvulsants, and phosphorus), reduced serum vitamin D levels, chronic kidney disease (estimated glomerular filtration rate (eGFR) < 60 mL/min/1.73 m^2^), renal calcium loss (hypercalciuria), and diseases of the gastrointestinal tract that interfere with calcium absorption (celiac disease, inflammatory bowel disease, and bariatric surgery) [3–6].

In the last decade, an increase in the prevalence of NPHPT has been observed, in addition to the recognition of clinical complications that were previously found in the classic and hypercalcemic forms of primary hyperparathyroidism (PHPT) [7–10]. The most common renal complication of hypercalcemic PHPT is calcification, which includes urolithiasis, with formation of stones in the calyx, pelvis, and ureters, and nephrocalcinosis, with diffuse deposition of calcium phosphate complexes in the renal parenchyma [11]. The investigation of this renal complication is recommended, even in asymptomatic patients [6]. Few studies have addressed the occurrence of this complication in NPHPT.

This study aims to investigate occult renal calcifications in patients with NPHPT and their associated risk factors.

## 2. Methods

### 2.1. Studied Population

Forty-five patients were selected from our endocrine outpatient clinic. Eleven patients with a history of urolithiasis or nephrocalcinosis were excluded. The diagnosis of NPHPT was based on the following criteria: serum PTH above the reference range (normal: 15–65 pg/mL) and serum calcium within the normal range (8.4–10.4 mg/dL), with values measured simultaneously and confirmed on at least 2 occasions, normal urinary calcium excretion (<4 mg/kg/24 h), serum 25(OH)D >30 ng/mL, eGFR >60 mL/min/1.73 m2, without intestinal diseases, and not on medications such as thiazide diuretics, lithium, bisphosphonates, or denosumab.

Patients who agreed to participate, after signing an informed consent form, answered a specific questionnaire and underwent a complete physical examination. Blood was collected after an overnight fasting for laboratory tests including PTH, 25(OH)D, total calcium, 1.25(OH)_2_D, albumin, phosphorus, urea, and creatinine, in addition to 24-hour urine calcium and urinary pH. Measurement of PTH and 25(OH)D was performed by electrochemiluminescence (Architect i2000 Abbott, USA), and 24-hour urinary calcium was assessed by calorimetry. Serum 1.25(OH)_2_D was measured by liquid chromatography tandem mass spectrometry. Albumin-adjusted calcium was used for this study and calculated according to the formula proposed by Figge et al. [12]:

(1) $$\begin{array}{c} \text{Adjusted Ca}=\text{total measured Ca}+\left[\text{0}.8\text{x}\left(4\;-\text{ albumin}\right)\right]. \end{array}$$

The reference range for adjusted calcium is 2.10–2.55 nmol/L (8.4–10.4 mg/dL), for PTH is 15–65 pg/mL, and for 1.25(OH)_2_D is 18–72 pg/mL.

The eGFR was calculated using the chronic kidney disease epidemiology collaboration (CKD-EPI) equation:

(2) $$\begin{array}{c} \text{eGFR}=\left(\frac{\text{mL}}{\text{min}}\times 1{\text{.}73\text{m}}^{2}\right)=175\text{x}{\left(\text{serum creatinine}\right)}^{-1154}\times {\text{age}}^{-\text{0}\text{.}203}\times \left[0.742\text{ if female}\right]\times \left[1\text{.}212\text{ if black}\right]. \end{array}$$

The patients underwent imaging studies for evaluation of renal calcification by computed tomography (CT) (Somatom Perspective 64 channels, Siemens, Germany) or ultrasound (US) with 3–7 MHz transducers (HD7 EX Phillips, The Netherlands). Half of the patients underwent CT imaging.

Patients were categorized into two groups based on the presence or absence of renal calcification, according to the result by the imaging method.

### 2.2. Statistical Analysis

Microsoft Office Excel was used for data collection and the IBM-SPSS version 25 program for statistical analysis. Descriptive statistics were expressed as absolute (N) and relative (%) frequency for categorical variables. For continuous variables, mean and standard deviation (SD) were used to describe variables with normal distribution and median and interquartile range for nonnormal distributions.

To compare the two groups (presence or absence of renal calcification) in relation to numerical variables, Student's *t*-test with equal variances for independent samples or the Mann–Whitney test was used. To determine the discriminatory power of the presence of kidney stones, a ROC curve was used for each variable, PTH, 1.25(OH)_2_D, and 24 h urinary calcium (mg/24 h), to calculate the area under the curve, test, and confidence interval for measure, sensitivity values, and specificity for the value that maximizes the sum of the sensitivity and specificity measures. The significance level used in interpreting the statistical test was 5%.

## 3. Results

Thirty-four patients were evaluated. Thirty-one were female (88.2% postmenopausal), with mean age of 67.97 ± 10.45 years and BMI of 26.17 ± 3.57 kg/m^2^. Laboratory results were as follows (mean ± SD) or median (interquartile range): serum PTH 99.80 (83.83–131.7) pg/mL, 25(OH)D 39.00 ± 8.88 ng/mL, 1.25(OH)_2_D 74.53 ± 26.37 pg/mL, albumin-corrected serum calcium 9.34 ± 0.62 mg/dL, phosphorus 3.45 ± 0.57 mg/dL, urinary pH 6.43 ± 0.50, and 24 h urinary calcium 127.50 (70.25–188.00) mg/day (Table 1).

Of the 34 patients, 9 (26.5%) had occult urolithiasis or nephrocalcinosis identified by CT or US. Two patients had nephrocalcinosis (5.9%) and 7 patients had urolithiasis (20.6%), with microcalculi size ranging from 1 to 9 mm. Among patients with urolithiasis, stones were identified as follows: 2 patients with a unilateral microcalculi (6 and 9 mm), 3 patients with bilateral microcalculi ranging from 5 to 7 mm, 1 patient with 3 unilateral microcalculi ranging from 1 to 2 mm, and 1 patient with 4 unilateral microcalculi ranging from 1 to 5 mm. All patients underwent investigation of renal calcification with US of the urinary tract, with 50% of the sample performing complementary CT. Renal calcifications were identified in 20.6% of patients who underwent US and in approximately 30% of those who underwent CT.

In comparison with patients without renal calcification, patients with calcifications had significant higher values for serum PTH levels (137.00 vs. 86.40 pg/mL, *P* = 0.001), 1.25(OH)_2_D (96.83 ± 20.00 vs. 62.36 ± 21.24 pg/mL, *P* = 0.005), and urinary calcium (160.00 vs. 116 mg/day, *P* = 0.037) and lower values for serum phosphate (3.13 ± 0.38 vs. 3.57 ± 0.59 mg/dL, *P* = 0.041) (Table 2). There were no significant differences between the groups regarding age, BMI, and waist circumference, serum 25(OH)D, eGFR, and urinary pH.

The ROC analysis showed that serum PTH, 1.25(OH)_2_D, and 24-hour urinary calcium had an area under the curve (AUC) of 0.850 (0.714–0.986, *P* = 0.003), 0.894 (0.737–1,000, *P* = 0.009), and 0.731 (0.554–0.909, *P* = 0.042), respectively. Serum PTH levels >101.30 pg/mL (sensitivity: 100%; specificity: 72%) and 1.25(OH)_2_D > 69.50 (sensitivity: 100%; specificity: 81.2%) were identified in all patients with renal calcifications. Twenty-four hour urinary calcium was also associated with the presence of occult calcification from 137 mg/day with sensitivity and specificity greater than 70% (Table 3).

## 4. Discussion

Renal calcification, which includes urolithiasis and nephrocalcinosis, is the most common complication of hypercalcemic PHPT [11, 13], but little is known about this complication in NPHPT. In this study, we identified the presence of renal calcification in 26.5% of patients with NPHPT, with a prevalence even higher than what has been reported in the literature when evaluating symptomatic urolithiasis in normocalcemic or mild hypercalcemic patients [14, 15].

With regard to the general population, urolithiasis has a lifetime prevalence of approximately 11% in men and 7% in women, according to data from the National Health and Nutrition Examination Survey (NHANES) [16]. In Europe, this prevalence is 5–9% [17], and a study in Brazil reported that the prevalence of kidney stones was 10.1% [18]. On the other hand, other studies that evaluated the prevalence of silent kidney stones in the general population did not exceed 3% rates [6, 19, 20].

The high rate of urolithiasis and nephrocalcinosis, even in asymptomatic patients with NPHPT, indicates that complications associated with PHPT can be an early event [21], and this is supported by current guidelines which recommend screening for occult renal calcification in all asymptomatic patients with PHPT [6]. Our data reinforce the importance of monitoring patients with NPHPT in order to identify early complications that were previously thought to be associated with hypercalcemic PHPT.

In this regard, Ejlsmark-Svensson et al. [11], using CT scans, demonstrated a 23% prevalence of renal calcifications with the same frequency of gender in patients with hypercalcemic PHPT (urolithiasis, 12%; nephrocalcinosis, 12%; both, 1%). On the same direction, the present study was able to demonstrate a similar prevalence of this complication in patients with NPHPT.

The similarity in the prevalence of urolithiasis between normocalcemic and hypercalcemic patients has been retrospectively reported by our group suggesting that NPHPT may not be a behaviour as an indolent condition [22].

In a recent study with 96 asymptomatic patients with hypercalcemic PHPT, a high incidence of occult urolithiasis (21%) was observed when active searching by renal imaging was done [23]. The higher frequency of urolithiasis in our study may be related to the use of CT scans in half of the patients, which enable a better performance for the identification of small calculi in comparison with ultrasound [24]. In the study by Tay et al., only 12.5% had CT scan, while 50% had US, 34.4% radiograph, and 3.1% magnetic resonance imaging. Participants who had renal CT images were more likely to have stones compared with images from other modalities (50% vs. 17%, *P* = 0.008) [23]. In the study by Starup-Lindel et al., using CT scans in 177 patients with hypercalcemic PHPT, the prevalence of renal calcification of 25.4% was similar to what we found in NPHPT in the present study [25].

US has lesser sensitivity (40% versus 97%) and specificity (84% versus 96–100%) when compared with CT for diagnosing renal stones, especially in the detection of stones smaller than 3 mm [26–28]. By including the tomographic evaluation in a significant portion of our casuistic, it was possible to detect stones from 1 mm and obtain more reliable prevalence data.

In the present study, higher serum PTH, 1.25(OH)_2_D, and urinary calcium were found in patients with renal calcifications compared to those without calcifications, and these parameters can be predictors of urolithiasis/nephrocalcinosis. This is in agreement with other studies in hypercalcemic PHPT [23, 25].

Of note, using US only, another study from our group was unable to show any differences in serum PTH or urinary calcium between NPHPT patients with or without kidney stones [10]. Although there was no statistically significant difference, this pilot study was carried out in a smaller sample which showed a trend towards higher PTH levels in stone formers patients. Serum 1.25(OH)_2_D was not measured in this study.

High serum PTH concentrations would promote greater stimulation of 1.25(OH)_2_D synthesis through renal hydroxylation of 25(OH)D, which, in turn, may increase filtered calcium load and predispose to hypercalciuria and renal calcifications [29], even with serum calcium in the normal range. In addition, as 1.25(OH)_2_D may influence calcium sensing receptor (CaSR) regulation and calcium channel expression, this may well be also a contributing factor for kidney stone formation as it would lead to a lower calcium resorption in the kidney and thus higher urinary calcium [30, 31].

Other factor that must be taken into account is some degree of PTH resistance in the kidney seen in patients with PHPT [31, 32]. Scillitani et al. [33] reported that patients with PHTP may have polymorphisms in the gene that encodes CaSR in the kidney and that it would modulate PTH actions increasing the risk of developing calcifications. These suggest that the formation of renal calcification in patients with NPHPT may be associated with other factors besides hypercalcemia and hypercalciuria [34, 35] or that there may be cutoff points within a spectrum of normality of serum calcium concentrations in which there is an increased risk for the development of kidney calcification.

The present study has some limitations. The cross-sectional design, the lack of a control group and ionized calcium measurements, although in at least two occasions, serum albumin-corrected serum calcium were below 10.3 mg/dL in all patients. 25(OH)D levels below 30 ng/mL were adopted as exclusion criteria, although some authors establish a threshold of 40 ng/mL for the diagnosis of NPHPT. Also, we do not have data regarding the patient's diet and urinary sodium and parameters that could influence the risk of hypercalciuria and renal calcifications. As strength, we were able to identify risk factors related to kidney calcifications in NPHPT, an area where research is urgently needed.

In conclusion, our data showed that occult renal calcifications are common in NPHPT and are associated with increased serum PTH, 1.25(OH)_2_D, and 24 h urinary calcium.

## Figures and Tables

**Table 1:** General characteristics of the study patients.

Age (years)	67.97 ± 10.45
Gender (% female)	31 (91.2%)
Body mass index (kg/m^2^)	26.17 ± 3.57
Abdominal circumference (cm)	86.43 ± 9.10
Serum PTH^*∗∗*^ (pg/mL)	99.80 (83.83–131.70)
Serum 25(OH)D (ng/mL)	39.00 ± 8.88
Serum 1.25(OH)_2_D (pg/mL)	74.53 ± 26.37
Serum calcium (mg/dL)	9.34 ± 0.62
Serum phosphorus (mg/dL)	3.45 ± 0.57
eGFR (mL/min/1.73 m^2^)	85.71 ± 13.14
Urinary calcium^*∗∗*^ (mg/24 h)	127.50 (70.25–188.00)
Urinary pH	6.43 ± 0.50

Data presented as mean ± SD or median (interquartile range). ^*∗∗*^PTH, parathyroid hormone; 25(OH)D, 25-hydroxyvitamin D*;* 1.25(OH)_2_D, 1.25-dihydroxyvitamin D; eGFR, estimated glomerular filtration rate.

**Table 2:** Laboratory data in NPHPT patients with and without renal calcifications.

	Renal calcifications (urolithiasis/nephrocalcinosis)				
Variable	N	Normal range	Present (*N* = 9)	Absent (*N* = 25)	*P*
Serum PTH^*∗∗*^ (pg/mL)	34	15–65	137.00 (104.10; 249.90)	86.40 (81.15; 113.80)	0.001
Serum 25(OH)D (ng/mL)	34	30–60	37.20 ± 6.92	39.65 ± 9.53	0.571
Serum 1.25(OH)_2_D^*∗∗*^ (pg/mL)	17	18–72	96.83 ± 20.00 (20.65)	62.36 ± 21.24 (34.06)	0.005
Serum calcium (mg/dL)	34	8.4–10.4	9.69 ± 0.67	9.22 ± 0.57	0.052
Serum phosphate (mg/dL)	34	2.5–4.5	3.13 ± 0.38	3.57 ± 0.59	0.041
eGFR (mL/min/1.73 m^2^)	34	>60	91.11 ± 13.61	83.76 ± 12.68	0.153
Urinary calcium^*∗∗*^ (mg/24 h)	34	<250	160.00 (122.50; 248.55)	116.00 (57.00; 183.50)	0.037
Urinary pH	34	5.5–7.5	6.62 ± 0.40	6.36 ± 0.51	0.195

Data are presented as mean ± SD or median (interquartile range). ^*∗∗*^PTH, parathyroid hormone; 25OHD, 25-hydroxyvitamin D; 1.25(OH)_2_D, 1.25-dihydroxyvitamin D.

**Table 3:** Predictors of occult renal calcifications in NPHPT patients.

Variable	AUC	*P* value	95% CI	Cutoff value	Sensitivity %	Specificity %
PTH (pg/mL)	0.850	0.003	0.714–0.986	101.30	100.0	72.0
Urinary calcium excretion (mg/24 h)	0.731	0.042	0.554–0.909	137.00	77.8	72.0
1.25(OH)_2_D (pg/mL)	0.894	0.009	0.737–1.000	69.50	100.0	81.2

AUC, area under the curve; CI, confidence interval; PTH, parathyroid hormone; 1.25(OH)_2_D, 1.25-dihydroxyvitamin D.

## Data Availability

The data used to support the findings of this study are available from the corresponding author upon request.
